# Outer Membrane Vesicles (OMVs) Produced by Gram-Negative Bacteria: Structure, Functions, Biogenesis, and Vaccine Application

**DOI:** 10.1155/2021/1490732

**Published:** 2021-03-24

**Authors:** Noemi Furuyama, Marcelo Palma Sircili

**Affiliations:** Laboratory of Genetics, Butantan Institute, São Paulo, Brazil

## Abstract

Gram-negative bacteria produce outer membrane vesicles (OMVs) with 10 to 300 nm of diameter. The contribution of OMVs to bacterial pathogenesis is a topic of great interest, and their capacity to be combined with antigens impact in the future to the development of vaccines.

## 1. Introduction

A broad range of microorganisms developed resistance to multiple antibiotics around the world, increasing the prevalence affecting human and animal health [1, 2]. Pathogenic bacteria can live in extremely hostile environments and use different mechanisms to circumvent stress conditions [3].

Gram-negative bacteria are major pathogens that develop resistance and cause distinct types of infections [1, 2, 4]. They inhabit almost all imaginable habitats but independent of the location; microbes have needed to develop tools to facilitate microbe-microbe, microbe-host, and microbe-environment interactions. These bacteria are capable of producing membrane vesicles (MVs) [5–7]. These MVs are spherical and frequently termed outer membrane vesicles (OMVs), microvesicles, exosomes, tolerasomes, agrosomes, and virus-like particles verified by electron microscopy [8–11]. All types of Gram-negative bacteria release extracellular membrane-bound vesicles like OMVs in a variety of environments, including planktonic cultures, liquid culture, solid culture, fresh and saltwater, biofilms, inside eukaryotic cells, and within mammalian hosts [6–8, 12]. Membrane vesicles were first observed over 50 years ago but generally considered insignificant largely ignored by microbiologists for several decades [13].

**Figure 1:**
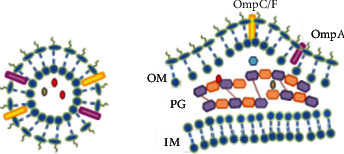
OMV production model—overview of Gram-negative envelope architecture in the context of OMV production.

The vesicle diameter is approximately 10 to 300 nm originating from the outer membrane (OM) and consists of lipids, proteins, lipopolysaccharides (LPS), phospholipids, DNA, RNA, proteins, inner membrane (IM), periplasm, OM, and other molecules from the periplasm between the IM and OM [5, 6, 8, 12, 14]. Therefore, several researchers concluded that OMVs are capable to provoke the immune system; then, they are recognized as promising agents to be used as vaccines [6] (see Figure 1).

Several studies have provided that OMVs have a multifaceted role both offensively as well as defensively [3, 5–7, 15–18]. Among their functions are improvement of bacterial survival by reducing levels of toxic compounds, neutralization of antimicrobial peptides conferring antibiotic resistance, aiding in the release of attacking phage, removal of stress products from the cell such as misfolded proteins, the formation of bacterial communities (biofilms), delivery of molecules, horizontal genes transfer, or enhancement of the immune response in host cells [3, 5–7, 15–17]. OMVs released from the envelope of pathogenic bacteria play an essential role in host-pathogen interactions including the establishment of a colonization niche, the transmission of virulence factors into host cells, and modulation of host defense [3, 5–7, 10, 14, 16, 17].

The research, over the years, has focused on the function of these vesicles, but only newly, the genetic and biochemical analyses led researchers to elucidate mechanistic aspects of OMV production [5, 13, 18]. Several studies demonstrated the complex cellular regulation of OMV production that is dependent upon many factors such as environmental conditions, pathogenicity, and the overall cellular metabolic state, varying according to species [5, 6, 12]. Before the discussion of these vesicles' biogenesis, it is important to consider the envelope architecture of the cell wall of Gram-negative bacteria.

In this review, we focused on the biogenesis of OMVs produced by Gram-negative bacteria and their use as a vaccine and drug delivery system. First, we describe aspects related to Gram-negative structures, antibiotic resistance, functions, and structure of OMVs.

## 2. Structures of Gram-Negative Cell Walls (See Figure 2)

The cell wall is a complex, multilayered structure that protects the contents of the bacteria cells preserving these organisms from their unpredictable and often hostile environment. It is responsible for the selective chemical barrier that defines cell shape and allows them to sustain large mechanical loads [14, 19–23].

Gram-negative bacteria envelope consists of two membranes that are chemically and structurally diverse: the inner membrane (IM) is a fluid phospholipid bilayer, peptidoglycan cell wall made up of repeating units of the disaccharide N-acetyl glucosamine-N-acetyl muramic acid, and the outer membrane (OM) with phospholipids in the inner leaflet and lipopolysaccharides (LPS) in the outer leaflet [8, 10, 14, 20, 23]. The periplasm is an aqueous compartment densely packed with proteins delimited by OM and IM [8, 10, 23, 24].

### 2.1. Outer Membrane (OM)

The OM is an asymmetric bilayer composed of lipopolysaccharides (LPS) in the external leaflet and phospholipids in the internal leaflet embedded with nonspecific porins and specific channels [21, 23]. The LPS molecule is responsible for the endotoxic shock associated with the septicemia produced by Gram-negative organisms, comprised of a basic lipid A (structure of phosphorylated N-acetyl glucosamine dimer with 6 or 7 fatty acids), core (R) antigen, R polysaccharide (a short chain of sugars), and antigen O attached to the core polysaccharide (repeating oligosaccharide subunits made up of 3 to 5 sugars) [21, 23, 25]. The sugar variation in the O side chain occurs between species of Gram-negative bacteria [25]. This membrane also has lipoproteins (Lpp) anchored via a covalently attached lipid moiety and b-barrel proteins termed outer membrane proteins (OMPs), such as porins like OmpF, OmpA, and OmpC [10, 23]. The OmpF and OmpC are responsible for the passive diffusion of small molecules, such as mono and disaccharides and amino acids across the OM [14, 20, 23]. OmpA is an abundant monomer that provides structural stability to the cell [23, 26]. OMPs at lower levels are responsible to transport large ligands such as Fe-chelates or vitamins such as vitamin B-12 [23]. The selective permeability barrier promotes by the porins limit diffusion of hydrophilic molecules bigger than about 700 Daltons [21, 23, 27]. The LPS bilayers block the passive diffusion of hydrophobic compounds [21, 23, 27]. The membrane porous permits diffusion for nutrition, waste removal, and transport of other molecules [14, 20, 23].

### 2.2. Peptidoglycan Cell Wall

The main structural features of peptidoglycan (PG) are a hetero-polymer made of linear glycan strands of alternating *β*1,4-linked N-acetylglucosamine (GlcNAc) and N-acetylmuramic acid (MurNAc) residues cross-linked by short peptides [28]. This compartment is a specific component of the cell wall localized outside of the cytoplasmic membrane of almost all bacteria, and it is essential to biosynthesis [29]. It is considered an exoskeleton-like because of the rigidity that envelope the cell defining the characteristics of bacteria [30]. This wall preserves the bacteria's shape limiting lysis from osmotic pressure and serves as a scaffold for anchoring other cell envelope components such as proteins. The hostile environments (e.g., antibiotic treatment) cause PG modifications in the peptides and sugar portions that protect bacteria during their growth [23, 29, 30]. These structural variations could lead to a weaker innate immune response, protection against predatory enzymes, evasion of the host immune system, and manipulation of the host metabolism to access carbon source [31–34]. The microorganisms developed quite mechanisms to alter the PG chemical structure to overcome specific threats to the cell wall [30].

### 2.3. Periplasm

The periplasm is a compartment densely packed with proteins that is more viscous than the cytoplasm, which contains a layer of peptidoglycan (PG) [8, 14, 23]. It is between the two bilayered membranes of the Gram-negative cell envelope. The periplasm has a dynamic flux that changes the varieties of macromolecules, reflecting the cell's metabolic and environmental status [14]. The proteins that inhabit this compartment possess functions to carry, chemotaxis, and envelope biogenesis. The periplasm can sequester degradative enzymes such as RNAse or alkaline phosphatase that are potentially harmful to Gram-negative bacteria [14, 23, 24].

### 2.4. Inner Membrane or Cytoplasmic Membrane

The inner membrane (IM) consists of a typical phospholipid bilayer that serves as an electrochemical barrier [8, 23]. The inner membrane also contains the receptors that sense the environment and the transport systems for nutrients and waste products. Bacteria lack intracellular organelles, and all the eukaryotic organelles are in the IM. This membrane possesses proteins that are responsible for energy functions, lipid biosynthesis, protein secretion, and transport [23].

## 3. Antibiotic Resistance

According to the WHO (World Health Organization), bacterial resistance is considered a public health problem community as well as health in the care facilities [1]. This resistance occurs when bacteria adapt to acquire the capacity to grow in the presence of antibiotics. The resistance to one specific antibiotic can lead it to a whole related class and spread rapidly and unpredictably through the exchange of bacteria genetic material between different strains affecting the treatment of too many infections and diseases [2].

In the hospital environment, the transmission of bacteria is too amplified because of the highly susceptible population. After the discovery and widespread use of antibiotics in the mid-20th century, many infections could be treated and cured [1]. However, the overuse and inappropriate application of antimicrobial agents contributed to the emergence of multidrug-resistant strains of bacteria [1, 2]. Besides that, antimicrobial resistance is driven by easy access through over-the-counter sales and sales via the internet [2]. Consequently, the rates of morbidity and mortality are increasing in the entire world, particularly in developing countries [1]. Many strains like *pneumococci*, *staphylococci*, *enterococci*, and *tuberculosis* are currently resistant to most of all antimicrobials [1]. Multiresistant *Klebsiella*, *E. coli*, *Pseudomonas aeruginosa*, and *Escherichia coli* are prevalent in many hospitals [1, 4, 21, 35].

In addition to the increased mortality and morbidity rate, the consequences of this problem can be severe, including prolonged illness, prolonged stays in hospital, risk of contamination of surgical devices, and increased costs. To counter the resistance, it needs long-term investment mainly for developing countries with financial, technical support, and the development of new vaccines or other immunobiological products [2], hence, are extremely important to develop new immunobiological to control infectious diseases. In this context, vesicles derived from pathogens have been studied for a long time to develop vaccine candidates against Gram-negative bacteria [7, 19, 36–40]. In this review, we focus on the outer membrane vesicles because these vesicles are a promissory strategy.

OMV contains many features necessary for an effective vaccine product: a native configuration for membrane surface antigens to elicit a humoral response, the potential to evoke a T-cell-mediated immune response, the presence of several pathogen-associated molecular patterns (PAMPs) to trigger the innate immune response, and appropriate size for efficient processing by antigen-presenting cells [7, 19, 36–41]. The potential for OMVs to mediate bacterial transformation, help bacterial communities to battle antibiotics, and spread antibiotic resistance genes [8, 18, 42]. In *E. coli*, the addition of OMVs or the use of a hypervesiculating mutant increased immediate resistance to the antimicrobial peptides polymyxin B and colistin 25 [3, 8]*. Acinetobacter baumannii* is also a relevant opportunistic pathogen in hospitals and shows severe drug resistance. OMVs can mediate to transfer carbapenem resistance through inhibition of membrane permeability, efflux pumps, drug inactivating enzymes, and drug target changes [8, 43]. Immunization with AbOMVs (*Acinetobacter baumannii* OMVs) produced high levels of antibodies that protected mice from infection by a drug-resistant strain [8, 43, 44]. *Stenotrophomonas maltophilia* treated with *β*-lactam antibiotic imipenem increased the OMV production, and the proteome analysis showed the presence of *β*-lactamase [45, 46]. The presence of this enzyme points to a protective role of vesicles when cells are in stress conditions [18, 46, 47].

## 4. Outer Membrane Vesicles (OMVs): Functions

There are many types of extracellular vesicles (EVs) correlated to the pathogenesis [7, 12, 47, 48]. OMVs are EV spontaneously released by Gram-negative and Gram-positive bacteria. However, the wild-type microorganism produces a small amount that is not enough to obtain the adequate quantity for large-scale production [47]. OMVs are spheroid particles approximately 10 to 300 nm pitched from the surface of the cell during all steps of bacterial growth, so the composition reflects the components of the outer membrane [6, 8, 12, 14]. They carry OMPs (outer membrane proteins), LPS (lipopolysaccharides), phospholipids, peptidoglycan, proteins (periplasmic, cytoplasmic, and membrane-bound), periplasmic components, nucleic acids (DNA, RNA), ion metabolites, and signaling molecules [6–8, 12, 14, 49].

The cargoes into vesicles enable them to drive specialized functions under environmental conditions like communication by quorum sensing (QS), biofilm formation, nutrient acquisition, antibiotic resistance, stress response, competition or defense against other microbes, environment, status of the microbial community, transfer of nucleic acids, horizontal gene transfer, toxin delivery, and virulence factors [3, 6, 10–12, 18, 19, 41, 45, 49–62].

Biofilm formation is a stress response and characterized by an exopolysaccharides matrix and other molecules such as proteins, lipids, and nucleic acids [6, 17]. Biofilm-derived OMVs play a role in signaling biofilm production via surface-associated DNA and also can mediate interactions within and external to the biofilm preventing damage against antibiotics and enzymes [6, 18, 41, 62–67]. Vesicles obtained by over time bacteria cultivation contain higher levels of antibiotics when compared with short time cultivation. The authors argue OMVs are operating as a reservoir for antibiotics in the biofilms [45].

The microbes are continuously competing in the environment. The OMVs produced by one bacterium can kill other microbes, even occurring among Gram-negative and Gram-positive bacteria [6, 68]. If the peptidoglycan hydrolases present in the OMVs are the same as another strain, the peptidoglycan layer cannot be cleaved by the enzyme. However, if OMVs fuse with cells of a nonself strain, then the enzyme can degrade the cell wall and kill the bacterium [6, 14, 68]. The vesicles of some bacteria have bacteriolytic enzymes capable of distinguishing between self and nonself cells, reaching or not the bacteria that surround it [6, 68, 69]. The autolysins and virulence factors or cytotoxins affect the host eukaryotic cells by OMVs [6, 68–73].

The secretion of OMVs is a mechanism to disseminate damaging virulence factors causing the bacterial infection [3, 5]. The bacteria can downregulate and deviate the host's innate immune system to establish an infection in a host. Thus, the OMVs can perform “offensive” and “defensive” functions. The vesicles can work as well as a virulence factor delivery mechanism and to aid the bacteria colonization in a hostile environment [3, 5]. OMVs can remove misfolded protein when the bacteria are exposed to chemical or physical stress, sequester and degraded the antibiotics, and decoy targets protecting cells by antibodies or phages [3, 5, 6, 41, 58, 74]. For example, OMV production increases the survival of bacterial cells treated with lytic bacteriophages [3]. The phage adhesion increases the vesiculation of OMVs and acts as an inducer of targets for the phages to protect the bacterium [3, 75]. OMVs can alleviate the stress caused by membrane-targeted peptide antibiotics, such as polymyxin B, by acting as decoy targets and transporting these molecules away from the cell [3]. Furthermore, the OMVs are fundamental for the survival of the bacterial community because the constant modification of the environment triggers the hypervesiculation [3, 6, 41, 58, 74].

OMVs could contribute to nutrient acquisition for bacterial survival carrying metal ions, degradative enzymes, and receptors [6, 41, 45, 69, 76]. There is intraspecies competition to obtain metal ions. According to Kulp and Kuehn, rare ions are concentrated into OMVs for consumption by bacterial cells in an adequate moment [6, 41]. Several reviews showed the importance of iron transported by OMVs during host invasion and for the transition of the bacterium from a planktonic to a biofilm lifestyle allowing the microorganism to thrive in this type of community [45, 60]. Lee et al. proteomics studies on OMVs showed the presence of different metal ion binding proteins [77]. Enzymes found in OMVs degrade complex biomolecules in the culture medium to make nutrients available [6]. Thus, these vesicles perform a relevant role in intraspecies nutrient transfer [6, 41].

Bacteria communicate in the microbial communities through small signaling molecules (AI = autoinducers), which constitute a complex regulatory network that controls the expressions of a variety of genes, including different phenotypes responsible for their virulent behaviors [78–80]. N-acyl homoserine lactones (AHLs) are the most studied signal produced by more than 70 species of Gram-negative bacteria of the Quorum Sensing (QS) system [78–83]. The *Pseudomonas* quinolone signal (PQS) of *Pseudomonas aeruginosa* promotes an increase in the curvature of the OM and consequently OMV formation [18, 78, 79, 84, 85]. Cooke et al. described a biophysical mechanism for this and recently showed it is operative in biofilms. They demonstrated that PQS-induced OMV production is highly dynamic during biofilm development. Interestingly, PQS and OMV synthesis are significantly elevated during dispersion compared to attachment and maturation stages. They show that purified OMVs can actively degrade extracellular protein, lipid, and DNA, and hypothesized that OMVs enhanced the production of PQS-induced OMVs during biofilm dispersion facilitates cell escape by coordinating the controlled degradation of biofilm matrix components [86]. Quorum sensing in the Gram-negative bacterium *P. aeruginosa* involves multiple signals including 3-oxo-dodecanoyl homoserine lactone (3OC12-HSL), butyryl homoserine lactone (C4-HSL), and PQS 2, 3. These molecules are part of a complex regulatory network and control the transcription of about 5% of all *P. aeruginosa* genes, including many genes involved in virulence. PQS is required for MV formation and the exogenous PQS mediates own packaging and the packaging of other quinolines into these vesicles. Cell-cell signaling and antimicrobial quinolone produced by *P. aeruginosa* are important for virulence, antibiotic resistance, and competition with other bacteria in the lung of patients affected by cystic fibrosis. Recent studies indicate that a bacterial cell-cell signal mediates the packaging of itself and other small molecules into MVs. The use of MVs to coordinate group behavior in a prokaryote is too important because they draw parallels to eukaryotic vesicle trafficking that are analogous systems to multicellular organisms [86, 87].

In summary, OMVs have a lot of roles. For example, they can contribute to bacterial survival by eliminating toxic compounds, neutralize environmental agents, and remove misfolded periplasmic proteins, establishment of a colonization niche, biofilm formation, drug delivery, and modulation of host defense and response [16, 88].

In the next item, we will discuss in more detail the delivery function promoted by OMVs.

## 5. Delivery Function

The bacterial OMVs have a multifaceted distribution system of interactions between interspecies and intraspecies, and the effects can be beneficial and harmful [89]. The cargoes into vesicles are transported at long distances protected from physical and biochemical stressors [47, 89, 90]. Vesicles export an assortment of biomolecules mainly proteins, virulence factors, LPS, DNA, enzymes, and toxins [18, 60–62, 91]. The OMV cargo promotes several advantages for pathogens. The proteins are insensitive to protease treatment and may arrive at their destination in a necessary concentration, as well as occur to other bacterial factors. Protein without a mechanism to self-direct adhesins or virulence factors can adhere to the OMV surface for transport to target. Vesicles enriched with lipopolysaccharide (LPS), and membrane-bound proteins exhibit immune-stimulatory capabilities facilitating the establishment of infection and inflammation [5, 12, 86, 91–93]. Moreover, these vesicles can transfer genes among bacterial species, small DNA fragments, autolysins to competing bacterial species, and drug-delivering synthetic nanoparticles used in vaccination [6, 16, 18, 47, 48, 68, 70].

In general, OMVs are released in increased amounts from pathogenic bacteria, suggesting that secretion is an additional virulence mechanism of pathogens. OMVs can take on both defensive and offensive tasks during infection. As defensively, they can be used to sequester antibiotics, bacteriophages, and antibodies; bind or degrade antimicrobial peptides; and bait antigens to distract the immune system. The potential of OMVs as offensive weapons is the ability to deliver virulence factors into host cells. Especially, OMVs produced and secreted by enterohemorrhagic *E. coli* (EHEC) have an advanced and simultaneous mechanism of secretion and delivery of bacterial virulence factors into host cells [94]. Enterotoxigenic *E. coli* (ETEC) is an important pathogen responsible for diarrhea and causes more than 700,000 childhood deaths due to diarrhea per year in third-world countries [1, 5, 8, 12, 91]. The enterotoxin heat-labile (LT) produced by ETEC disrupts electrolyte balance in the gut endothelium is associated with vesicles and contributed to pathogenicity [91]. Kesty et al. showed that pathogenic ETEC utilizes vesicles to deliver LT that catalyzes internalization of microorganism vesicles into the host cell mucosal layer inducing the infection, locally, or away from the site of the colonization. Aside from toxic compounds, vesicles containing DNA were verified in *Pseudomonas aeruginosa*, *Neisseria gonorrhoeae* with linear and circular DNA resistant to enzymatic hydrolysis, *Escherichia coli* O157:H7, *Haemophilus influenzae*, and other several bacterial species [70, 95, 96]. Horizontal transfer of genes was mediated by vesicles isolated from *Escherichia coli* O157:H7 pathogen that facilitate the transfer of genes to *Salmonella enterica*, and DNA fragments and was observed among bacterial species, and it can be correlated by genes encoding virulence factors and antibiotic resistance [70]. Lin et al. (2017) verified that iron ions are essential in every life process involved in metabolism, proliferation, and pathogenic bacteria to cope with nutritional challenges, developing many effective strategies to scavenge metal iron from the environment. In *Mycobacterium tuberculosis*, membrane vesicles are involved in iron acquisition [97, 98]. *P. aeruginosa* presented the gene TseF, which facilitates the delivery of OMV-associated iron to bacterial cells by directly interacting with the iron-binding *Pseudomonas* quinolone signal (PQS) [97, 99]. *Actinobacillus actinomycetemcomitans* causes periodontal diseases, and membranous vesicles carry several virulence-associated proteins as a leukotoxin [100].

Many antibiotics available are ineffective against intracellular infections, mainly because of difficulty in penetrating or decreasing the activity of the drug [18, 101]. The use of OMVs as a delivery system is advantageous because they are considered natural agents produced by bacteria, are quickly encompassed by eukaryotic cells, and are capable of delivering active drugs [101]. *Pseudomonas aeruginosa* untreated with antibiotics releases normal vesicles (n-MVs), however, when treated by gentamicin produces gentamicin vesicles (g-vesicles) [101, 102]. These g-MVs vesicles were able to kill pathogen cultures, including a gentamicin-resistant *P. aeruginosa* strain, probably because g-MVs (combined with antibiotic and autolysin) allowed to overcome the permeability barriers of the surface and release antibiotic directly into the bacterial periplasm [101]. *Escherichia coli* outer membrane vesicles coated with synthetic nanoparticles with carrying function demonstrate the capability to generate a high immunological response showing that this technology is a great promise for designing effective antibacterial vaccines [47, 103].

Focusing on the medical field, the OMVs have many characteristics and functions desirable of drug delivery vehicles [18, 48, 104, 105]. The small size permits them to evade immediate capture and clearance by the host immunological system. The vehicle must not be immunogenic and inflammatory, so to achieve this, different approaches will need to develop OMVs as drug delivery vehicles. For example, LPS's main component of the vesicles is a potent activator of immune cells that is advantageous in the development of vaccines or adjuvants.

However, it causes a systemic and strong inflammatory response that is undesirable in drug delivery applications. Genetic engineering minimizes this response [48]. The incorporation of molecules into vesicles can be realized by two methods, before or after their isolation [48, 106]. The first approach is a method used for molecules such as RNA or aminoglycoside antibiotics incorporated after isolation, because to loaded these molecules during the cultivation of bacteria is a complex process [48, 105, 107]. The small RNAs can treat diseases in which specific genes are overactive, such as cancer [105]. The techniques to load the vesicles with the drug using the first method are osmotic gradients, electroporation, ultrasonication, or enhancing the membrane permeability using cell-penetrating peptides or chemical transfection [48, 106]. The second approach is OMVs loading hydrophobic drugs and hydrophobic compounds during bacterium cultivation, for example, curcumin an anti-inflammatory drug [103, 104]. In vitro, this drug inside the OMVs showed an increase in solubility and stability, and in vivo, it increased the bioavailability. Curcumin injected promoted protection against septic shock induced by LPS in mice. Although OMVs are considered promising drug delivery vehicles, it is necessary to develop techniques and approaches for obtaining them in large quantities. Also, a determining factor for effective therapeutic action is an optimization of biodistribution methods in the patient's organism is required [105, 106].

The enforcement of OVMs as a delivery system for vaccination application we will discuss in more detail.

## 6. Biogenesis

**Figure 3:**
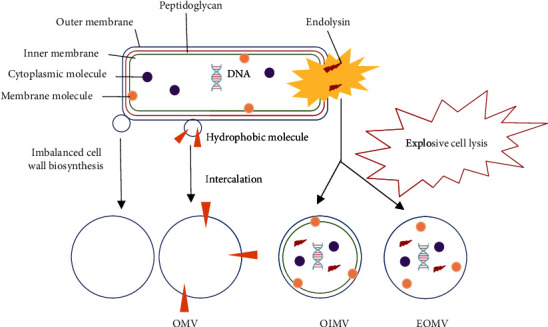
Gram-negative bacteria have two main routes for vesicle formation: blebbing of the outer membrane and explosive cell lysis producing outer membrane vesicles (OMVs) and occurs as a result of cell envelope disturbances. Explosive cell lysis is triggered by endolysin, which degrades the cell wall of the peptidoglycan, generating the inner-outer membrane vesicles (OMVs) and the explosive outer membrane vesicles (OMVs).

The biogenesis of OMVs is a budding process of the outside of the outer membrane. There is much evidence that vesiculation in Gram-negative bacteria is not a passive process. Nevertheless, it is sophisticated machinery for the secretion of various main biological molecules [6, 11, 16, 38, 41, 45, 76]. The wild-type bacteria naturally shed OMVs at low concentration but is not enough to achieve a reasonable quantity to large-scale production for pharmaceutical and biotechnology applications [16, 41]. Their hypervesiculation occurs by a variety of factors, such as quorum sensing, temperature stress, altered nutrients, oxidative stress, antibiotics, and envelope stress [16, 58, 108, 109]. OMVs can be endowed with several abilities using genetic modification like as deliver proteins, stimulate the body to generate immune protection, increase the bacterium vesiculation, reduce endotoxin with less reactogenic LPS structures, display antigens on their outer membrane generating a recombinant OMV (rOMVs), produce a new generation of vaccine and, and function as an adjuvant activity [110]. There is no evidence of energy expenditure during OMVs biogenesis; however, probably, it is necessary to determine which biomolecules will be carried by OMVs [6, 41]. A recent review divided the bacterial membrane vesicles from Gram-negative bacteria into outer inner membrane vesicles (OIMVs), explosive outer membrane vesicles (EOMVs), and traditional OMV [111] (see Figure 3). The integrity of the outer membrane is not compromised when the OMVs are produced spontaneously. While when are produced artificially their lumen contents differ from naturally OMVs [110, 112].

There are some models proposed for OMV mechanism formation. The first model hypothesis is about the loss or relocation of covalent linkages defect between the outer membrane and the underlying peptidoglycan layer [6, 41, 112, 113]. When it occurs, a faster growth rate of the outer membrane than the underlying cell wall allows the outer membrane to protrude and finally generate OMV [6, 41].

The interaction between the outer membrane and turgor pressure is the second model. The accumulation of peptidoglycan fragments or misfolded proteins in the periplasmic space exerts a turgor pressure and induces the protuberance of the outer membrane [6, 111–116]. For example, in *Escherichia coli*, the outer membrane tolerates 3 atm of turgor pressure, and peptidoglycan fragments can exceed this limit and force vesicles to be released [115, 117]. In *Pseudomonas aeruginosa*, the depletion of Opr86, which has a role in outer membrane protein (OMP) assembly, resulted in increased expression of the periplasmic serine protease (MucD) production in hypervesiculation. The misfolded OMPs in the periplasm induced the OMV biogenesis [116].

The third model (bilayer couple model) defends the hypothesis of membrane increased curvature. *Pseudomonas aeruginosa* has Pseudomonas quinolone signal (PQS) and uses this extracellular signal to communicate and coordinate social activities. This quorum sensing molecule also mediates its packaging and transport by stimulating outer membrane vesicle (OMV) formation that serves to traffic this molecule within a population [118]. PQS stimulates the anionic repulsions between LPS molecules resulting in membrane blebbing [41, 87, 100, 114, 115, 119]. The PQS is limited by the fact it is exclusive to *P. aeruginosa*, therefore, species-specific [41, 112, 120].

In the fourth model, when *vacJ* and/or *yrb* genes are silenced or deleted, phospholipids (PL) accumulate in the outer leaflet of the outer membrane resulting in an asymmetric expansion of the outer leaflet and promotes the budding of the outer membrane to form an OMV [112, 117]. OMVs derived from the PL transporter mutants (∆*vacJ* and/or ∆*yrb*) contain higher PL levels compared with wild-type OMVs. The hypervesiculation observed in the wild-type as well as in the mutant strains secretes vesicles with a similar size distribution, indicating the same amount of PLs in the inner leaflet of the vesicle membrane [112]. Thus, the PL transporter genes are highly conserved. The hypervesiculation by mutants appear in a variety of Gram-negative bacteria like *Haemophilus influenzae*, *Vibrio cholerae*, and *Escherichia co*li [111, 117]. This model would be perfect as a mechanism of OMV secretion by Gram-negative bacteria, however, does not fully explain why the OMV contains DNA inside the bacterial inner membrane. Therefore, more studies are needed to explain the mechanism of OMV biogenesis [112].

Another study with *Escherichia coli* exposed to damaging stressors results in the activation of stress responses that are compartmentalized and managed by specialized systems. The activities of chaperone and protease of *E. coli* DH5*α* mutant (MK11F26 *degP*::Tn5 lacking DegP) submitted by elevated temperatures are damaged, and, consequently, misfolded proteins are formed and eliminated through OMVs. This mutant exhibits a strong overvesiculation phenotype compared to strain complemented with a *degP* plasmid (pCS20) that showed >100-fold reduction OMV formation [114].

None of the models mentioned convincingly explain how DNA is present in OMVs. Another new formation mechanism route argues the enzymatic action of endolysins [111]. The *P. aeruginosa* bacterium submitted to a stress condition has its DNA damaged. So the endolysin expression is induced and triggering the degradation of the peptidoglycan layer [53, 111]. Consequently, the cells explode (phenomenon named explosive cell lysis), and the remaining membrane fragments round-up and self-assemble into OMVs named EOMVs [111]. These formed vesicles also carry endolysins and then may lyse other cells, which turn to generate more vesicles [68, 111, 121]. Although this route requires the death of a small subpopulation of cells, it provides a benefit to the surviving population. For example, some antibiotics induce the SOS response, cell lysis, and vesicle formation in lysogenic bacteria. The remaining bacterial population becomes protected by the OMVs and can neutralize the environmental agents that target the outer membrane, including phages, antibiotics, and eukaryotic host to defense factors [3, 111, 119].

In *C. violaceum*, the CviI/CviR QS system adjusts the OMV release rate by activating both violacein biosynthesis and the VacJ/Yrb system in the stationary phase, two OMV biogenesis pathways with an inverse role in vesiculation. Our data indicating that mutation or downregulation of *vacJ*/*yrb* genes caused hypervesiculation to support the emerging notion that bacteria control the OMV release rate by regulating the VacJ/Yrb pathway (Roier et al., 2016). Recent reports have found that several input signals, such as iron limitation, bile salts, and host entry, cause downregulation of the *vacJ*/*yrb* genes resulting in an increased OMV release that modulate bacterial adaptation to host conditions. Therefore, future works addressing the role of OMVs in *C. violaceum* virulence will be of particular interest [122].

*Vibrio cholerae* showed another mechanism for OMV formation. It assembles flagella that are surrounded by a sheath derived from the outer membrane. Membrane blebs carrying LPS are along the sheathed flagella that are released when the flagella rotate [22, 87, 116]. Flagellar motility is a relevant virulence trait, suggesting that the phenomenon of flagellar-mediated LPS release through OMVs and may be widespread among bacteria [112].

McBroom et al. 2006 [74] suggested there are many genes responsible for the overproduction of OMVs related to peptidoglycan synthesis, OM proteins (OMPs), and the sigma E stress response pathway. The OMV biogenesis is not only a stress response but also a vital physiological process found in all Gram-negative bacteria [6, 74]. The “engineer designer” of these vesicles through genome engineering could generate bacterial “factories” that enable optimization of OMV production required to develop an excellent biotechnology product. Using the same strategies possibly remodeled the lipid A structures to eliminate the toxicity, reprogramed the protein cargo, and decorated the interior and exterior of OMVs with specific combinations such as unique antigens, antibodies, receptors, receptor ligands, and/or enzymes [6, 16, 123].

## 7. OMVs and Vaccine Application

Today, vaccine development is the most active research field in biomedical sciences [124]. Prophylactic vaccination eradicated smallpox, rinderpest, and poliomyelitis [125–128]. The continuous development of vaccines is required to prevent the emergence of new infectious [129]. Despite vaccination saving many lives by preventing infections, diseases remain a major source of mortality worldwide [129]. There are many reasons for the increasing demand for the development of new vaccines. First, infectious diseases show antigenic variation reducing their potency of vaccines; second, bacterial antimicrobial resistance; and third, the introduction of single serogroup-specific vaccines for a specific disease causes another serogroup emergence [56, 129].

The vaccine platforms can provide enhanced safety, productivity, and simplicity to obtain the most suitable product [90, 129]. The platform is aimed at promoting high and lasting host immune antibodies against specific antigens, a standard innate response, protection against different diseases induced by displaying different antigens, and reducing significantly the time to market [47, 90, 129]. Bacterial outer membrane vesicles carrying antigens are an important candidate as a vaccine platform. Moreover, the application of bioengineer technology in the heterologous antigens into vesicles will enable to induce a high immune response [47, 129]. Summing up, the stimulating innate immunity and promoting adaptive immune responses attributed to OMVs is characterized due to 3 key features [125]. First, they carry surface-associated antigens. Second, they quickly phagocytize antigen-presenting cells and carry many pathogen-associated-molecular patterns (PAMPs) [125].

Bielig et al. propose that the regulation of the peptidoglycan content in OMVs is used by bacteria to evade Nod-like receptors- (NLR-) mediated immune detection in the host. Quorum sensing plays an important role in this process in *V. cholerae* but likely this is used also by other bacteria. OMVs deliver bacterial virulence factors, and recent studies revealed that OMVs are also critical to delivering both membranes bound and soluble luminal (i.e., periplasmic) pathogen-associated molecular patterns (PAMPs) to the host cell, as a result, occurs the activation of membrane-associated and intracellular pattern-recognition receptors (PRRs). By understanding how bacteria control PAMP, the composition of OMVs will give us the tools to manipulate OMV production and optimize its immunogenic properties. This likely will help to boost the use of OMVs as future vaccine candidates [130]. These data provide evidence for the physiological relevance of bacterial MVs in cell-cell signaling and justify future works to a better understanding of the regulation of prokaryotic social activities.

OMVs are at the interface between traditional and new methods of vaccine production and represent a feasible opportunity to control various infectious diseases, such as nosocomial infections, enteric diseases, tuberculosis, meningitis, and whoop coughing that remain a health problem in children and young adults [36]. Vesicles presented advantages as a vaccine candidate because they are nonreplicative particles so cannot cause the disease, work as self-adjuvating, small size and particle shape facilitate the distribution throughout the body, highly stable at varying temperatures, elicit long-term memory responses, and induce both humoral and cellular mediated immune responses against OMV-presented antigens [56, 125, 131–135].

Currently, licensed vaccines based on OMV use detergent extraction to reduce the lipopolysaccharide (LPS) which is very toxic [36, 129]. The VA-MENGOC-BC®, MenBVac®, MeNZB®, and Bexsero® are examples of OMV-licensed vaccines obtained using deoxycholate detergent extraction of the bacterial membranes [36, 136, 137].

Therefore, OMVs can prevent bacterial infections. Studies with *E. coli*-derived OMVs had a high protective effect. It was efficiently prevented from bacterium-induced lethality and induced systemic inflammatory response syndrome via Th1 and Th17 cell responses [47, 84, 138]. The OMVs of *Campylobacter jejuni* are an alternative for the delivery of proteins into host cells that confer cytotoxic activity and induce a host immune response in intestinal epithelial cells [47, 139]. *Pseudomonas aeruginosa* outer membrane vesicles activate a significant IL-8 proinflammatory response in lung epithelial cells and induced pulmonary inflammation via increasing chemokines and cytokines in the mouse lungs and mouse alveolar macrophages in a rodent model [47, 76]. The inflammatory responses induced by OMVs compared to live bacteria indicated that OMVs have a similar ability to produce innate immunity [47, 140]. Mice immunized with *Salmonella* OMVs developed robust B and T-cell responses, moreover, stimulated IFN-g production by a large proportion of CD4_T cells in mice previously infected with these bacteria [141]. *Bordetella pertussis*-derived OMVs are protected against infection from pertussis in a mouse model. The effect was comparable to the whole-cell formulation of vaccines (WHO reference strain). In clinical research, of multicomponent Men B vaccine (4CMenB), containing three broadly conserved surface-expressed recombinant antigens and specific OMV provided broad protection against circulating heterologous strains of MenB. This formulation has proven to be immunogenic in adults, adolescents, and young infants and the most susceptible age groups [47, 142]. OMVs derived from *Klebsiella pneumoniae* are important secretory nanocomplexes that elicit a potent inflammatory response [47, 143]. The mice immunized with vesicles of V*ibrio cholera* isolated from enteric pathogens (detergent or free-detergent) showed immunogenic and protective results [144, 145]. OMVs obtained by *E. coli* strains, both enteropathogenic (EPEC) and enterotoxigenic (ETEC) strains, showed a high specific antibody response and heterologous cross-reactivity among them [36]. Vesicles derived from nonpathogenic mycobacteria *Mycobacterium smegmatis*, which have high levels of genomic and antigenic homology with *Mycobacterium tuberculosis* (MTB), induced cross-reactive immune responses against MTB antigens at the cellular and humoral levels in mice [146].

The OMVs have a lot of applications, and to enhance their yield, engineering modifications are necessary [124]. Bioengineering the OMVs, it is possible to modify different bacterial species that naturally display an array of biological effects and targeting specificities to produce vesicles with specific characteristics using relatively simple molecular techniques [48, 56]. The genetic manipulation allows packaging of recombinant epitopes including signal molecules for cell-specific targeting, excluding undesired signals, modifying toxic components, genetically engineering the vesiculating strain [48, 56, 90, 124, 147]. The application of gene, pathway, and genome engineering will enable the hypervesiculation of OMV to create bacterial “factories” required for the realistic biological application [16, 56, 124].

**Figure 4:**
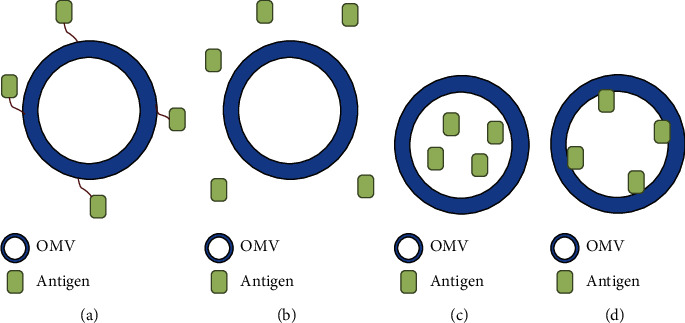
Design of antigen decoration on OMVs. (a) and (b) show surface-exposed antigens on the vesicles; (c) and (d) show the antigens as luminal cargo of OMVs.

The antigens, proteins, specific ligands, and antibodies can be displayed either inside the OMV or on the surface enriched with specific ligands, such as antibodies and antigens [48, 124, 129]. The location (design) of molecules is important for provoking the desired immune response. These heterologous antigens can be presented with or without surface exposure, produced by the bacterium (endogenous antigens), and combined in a later production stage (exogenous antigens) [129] (Figure 4). The immune system modulates according to the design of the OMV activating humoral and/or cellular response [37, 129]. The first design is endogenous loading of surface-exposed antigens based on the expression of proteins on the outer membrane, however, many studies showed low yield or are only suited for small proteins or parts thereof [129, 148]. Kim et al. [149] constructed protein fused with several heterologous proteins, including GFP with a five residue glycine linker to the C-terminus of the pore-forming cytotoxin ClyA, that were efficiently transported across the inner membrane to the outer membrane of *E. coli* [149]. Endogenous loading of antigens to the OMV lumen is a second approach, and there are different techniques. McBroom and Kuehn [114] demonstrated that it is possible to enrich specific proteins in the OMV lumen, adding a misfolded outer membrane protein sequence to the periplasmic cytochrome b562 [114]. Kesty and Kuehn [150] bioengineered antigens targeted to the lumen of vesicles, they fused to the twin-arginine (Tat) signal sequence to produce *E. coli* OMVs with GFP in their lumen, and this pathway transferred folded proteins over the cytoplasmic membrane. The GFP protein in the lumen of the vesicles was stable, therefore, protected against the action of proteinases [150].

Another method for luminal protein expression obtained success. Bartolini et al. (2013) and Fantappie et al. (2014) fused proteins in the periplasmic side with outer membrane protein (OmpA) to secretion signals or periplasmic proteins. OmpA truncations or deletions resulted in a blebbing phenotype that was inferred from its differential immunoprecipitation and resistance to proteolytic degradation [60, 129, 151, 152]. The exogenous loading of surface-exposed antigens is the third method, the antigens introduced after OMV mass production [129]. Alves (2015) loaded phosphotriesterase (PTE) fused by OmpA through SpyCatcher/SpyTag (SC/ST) bioconjugation system [153]. OmpA is a highly expressed porin protein present in the bacterial outer membrane and subsequent OMVs. The PTE breaks organophosphates making them less toxic. Exposure to this molecule most commonly causes convulsions and death via asphyxiation [153].

Exogenous loading of antigens to the OMV lumen is another approach to loading antigen after OMV mass production too. The vesicles are opened and closed without permanent damage. There are studies of loading smaller molecules into extracellular vesicles (EVs) passive or actively loading using electroporation, saponin-treatment, extrusion, or dialysis [154]. Gujrati et al. (2014) encapsulated siRNA into *E. coli* OMVs targets kinesin spindle protein, which is upregulated during tumor and rapidly growing cells [104].

As we verified, bioengineering can design OMVs for direct use as a vaccine because it can induce excellent both humoral and cellular immune responses [129]. Furthermore, the suitable vesicle design can reduce the toxicity of LPS that causes severe side effects in the traditional vaccines, detoxifying this molecule [16, 38, 90, 112, 124, 129]. Recently, Watkins et al. (2017) showed recombinant *E. coli* strain constructed only with lipid portion of LPS IVa instead of complete LPS. This recombinant OMV (rOMV) showed attenuated pyrogenicity and high levels of immunogenicity, moreover, promotes a balanced Th1/Th2 humoral response [155]. Another way to reduce the toxicity is the expression of heterologous glycan antigens instead of antigenic proteins [124, 129]. The recombinant polysaccharide conjugated with outer membrane vesicles resulting in glycol-engineered outer membrane vesicles (geOMVs) that can effectively deliver pathogen-mimetic glycotopes to the immune system [129, 153].

The combinations of different OMVs and their capacity to be combined with antigens may have a relevant impact in the future on the development of vaccines against pathogens [36]. Thus, with the construction of mutant strains with overexpressed protein vaccine antigens naturally inserted into the vesicles, a high number of vaccine candidates with these characteristics will improve the yield, immunogenicity, and safety profile of the production OMVs, in a few years [36, 156].

## 8. Conclusions and Future Directions

Persistent use of antibiotics provoked the emergence of multidrug-resistant (MDR) and extensively drug-resistant (XDR) bacteria. Extended-spectrum b-lactamase (ESBL) and carbapenemase-producing Gram-negative bacteria emerged as a relevant therapeutic challenge. There is a resurgence of classical bacterial diseases and emerging of new bacterial and viral diseases because of the inefficiency of antibiotics. *Enterococcus faecium*, *S. aureus*, *K. pneumoniae*, *A. baumannii*, *P. aeruginosa*, and *E. coli* (ESKAPE pathogens) are the six nosocomial resistant bacteria that seriously threaten the lives of patients [43]. The exploration and application of OMVs as a vaccine platform include the optimization of the appropriate innate and adaptive immune responses by either removing or inserting specific components, which will be individually evaluated for each disease application [38].

The OMVs have diverse functions and are fundamental for the survival of Gram-negative bacteria that have a multifaceted function that influences bacterial ecology. Therefore, with the knowledge of the ecological role, biogenesis, genetic basis, and the exact pathway of OMVs stimulation, we can get enhanced yield to obtain the best product to combat the bacterial pathogens. The high-yield OMVs for the preparation of vaccines are a prerequisite to developed good OMV-based vaccines. The amount of OMVs produced is a response to growth conditions, stress factors, and growth phases of bacterial cultures. There are many genes involved in increasing or decreasing OMV production. *Pseudomonas* naturally produces higher yields of OMVs than other bacteria, so expressing antigenic proteins of interest in a species with a higher yield of OMVs may be advantageous [6].

There are broad research lines related to the development of OMVs vaccines, and one of them is the QS systems. There are promising targets for developing new anti-infective compounds based on the regulatory function of these systems in the pathogenesis of bacteria to control the spread of antibiotic-resistant [157]. QS-controlled expression of virulence genes in *E. coli* is mediated by signal molecules, such as indole, Acyl homoserine lactones (AI-1), Furanosyl diester (AI-2), and aromatic compounds, such as AI-3, epinephrine, and norepinephrine that control the type III secretion system which is a virulence determinant required for the formation of characteristic attaching and effacing (A/E) lesions [158].

OMVs proved to be a flexible vaccine production platform and very complex structures that contain immune stimulators (e.g., LPS, proteins, and DNA) and antigenic molecules delivered to immune-competent cells of the immune system [36, 124, 159]. Therefore, OMV has an intrinsic adjuvant effect overloaded antigens from bacteria, but also over heterologous antigens that can be incorporated or combined in a single formulation, the immune-stimulating properties of the vesicle can be engineered, and the toxicity can be reduced [36, 112, 124, 159]. Moreover, the versatility to enable administration via the mucosal or parenteral route offers a significant choice. The adjuvant potential and increased knowledge in the design of OMV over the last few decades will also enable the future development of the next generation of novel vaccine formulations [36, 124, 159].

The progress in the production of these vesicles is excellent, but it is not easy to control the vesiculation because there are many differences between batches during the fermentation process. Therefore, bioengineering is fundamental to obtain more sophisticated OMV eliminating problems like LPS toxicity, large-scale production, biological engineering, load heterologous proteins, improving safety, and reducing costs [16, 90].

The availability of high throughput like proteomics genomics, lipidomics technologies, automation in microbiological techniques, and support from bioinformatics makes the exploration more practical to help in preparing engineered OMVs [6]. The use of biotechnology to design OMVs as carriers of vaccine preparations is the most promising tool to develop new vaccines [160]. Moreover, with genetic modifications, OMVs are able to perform multiple functions carrying molecules [90]. OMV is a bionanoparticle with many capabilities and can be applied in many fields such as immunology, diagnostics, clinical medicine, and others [90]. With the continuous studies on OMV-based nanotechnology, it will be possible to develop a powerful immunobiological tool.

## Figures and Tables

**Figure 2:**
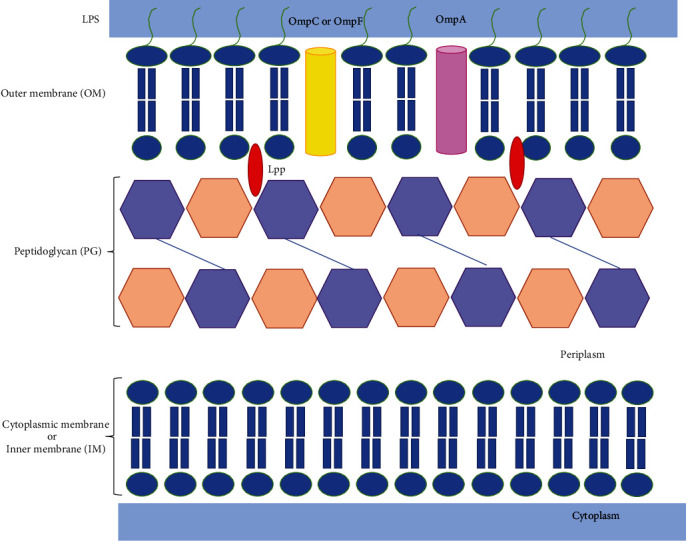
The cell envelope of Gram-negative bacteria consists of two membranes, the outer membrane and the cytoplasmic membrane. The cytoplasmic membrane is composed of a phospholipid bilayer, whereas the outer membrane comprises an interior leaflet of phospholipids and an exterior leaflet of lipopolysaccharide (LPS); LPS is composed of lipid A, the core oligosaccharide, and O antigen. In between the two membranes is the periplasmic space, which contains the peptidoglycan (PG) layer and periplasmic proteins.
